# Risk of African swine fever virus transmission among wild boar and domestic pigs in Poland

**DOI:** 10.3389/fvets.2023.1295127

**Published:** 2023-11-06

**Authors:** Kim M. Pepin, Tomasz Borowik, Maciej Frant, Kamila Plis, Tomasz Podgórski

**Affiliations:** ^1^National Wildlife Research Center, USDA, APHIS, Wildlife Services, Fort Collins, CO, United States; ^2^Mammal Research Institute, Polish Academy of Sciences, Białowieża, Poland; ^3^Department of Swine Diseases, National Veterinary Research Institute, Puławy, Poland; ^4^Department of Game Management and Wildlife Biology, Faculty of Forestry and Wood Sciences, Czech University of Life Sciences, Prague, Czechia

**Keywords:** African swine fever, wild boar, domestic pigs, surveillance, wildlife-livestock

## Abstract

**Introduction:**

African swine fever (ASF) is a notifiable disease of swine that impacts global pork trade and food security. In several countries across the globe, the disease persists in wild boar (WB) populations sympatric to domestic pig (DP) operations, with continued detections in both sectors. While there is evidence of spillover and spillback between the sectors, the frequency of occurrence and relative importance of different risk factors for transmission at the wildlife-livestock interface remain unclear.

**Methods:**

To address this gap, we leveraged ASF surveillance data from WB and DP across Eastern Poland from 2014–2019 in an analysis that quantified the relative importance of different risk factors for explaining variation in each of the ASF surveillance data from WB and DP.

**Results:**

ASF prevalence exhibited different seasonal trends across the sectors: apparent prevalence was much higher in summer (84% of detections) in DP, but more consistent throughout the year in WB (highest in winter with 45%, lowest in summer at 15%). Only 21.8% of DP-positive surveillance data included surveillance in WB nearby (within 5 km of the grid cell within the last 4 weeks), while 41.9% of WB-positive surveillance samples included any DP surveillance samples nearby. Thus, the surveillance design afforded twice as much opportunity to find DP-positive samples in the recent vicinity of WB-positive samples compared to the opposite, yet the rate of positive WB samples in the recent vicinity of a positive DP sample was 48 times as likely than the rate of positive DP samples in the recent vicinity of a positive WB sample. Our machine learning analyses found that positive samples in WB were predicted by WB-related risk factors, but not to DP-related risk factors. In contrast, WB risk factors were important for predicting detections in DP on a few spatial and temporal scales of data aggregation.

**Discussion:**

Our results highlight that spillover from WB to DP might be more frequent than the reverse, but that the structure of current surveillance systems challenge quantification of spillover frequency and risk factors. Our results emphasize the importance of, and provide guidance for, improving cross-sector surveillance designs.

## 1. Introduction

Understanding the risks of pathogen transmission across the wildlife-livestock interface is key to mitigating threats to human health (1), food security (2), and endangered wildlife (3). Pathogen transmission from wildlife to domestic hosts or the reverse results from a combination of epidemiological, ecological and behavioral drivers of pathogen pressure in the reservoir host, pathogen exposure in the receiving host, and structural barriers to contact at the interface between them (4, 5). Force of infection at the interface will depend on the pathogen prevalence in the donor host population, contact rate between the reservoir and recipient host, and probability of infection given contact (6). On the donor host side, pathogen pressure is generated by the interaction of host ecology (population distribution, connectivity, and density, host movements and contact structure) and pathogen ecology (routes of transmission, survival in the environment) which determine prevalence, persistence and spread (4, 7). If wildlife and domestic host populations have similar susceptibility and transmission ability to a particular pathogen, transmission between the two can be bidirectional (8–10), yet with distinct disease dynamics in each population due to differences in ecological context. Surveillance systems that include data from each host population jointly are important for understanding transmission risk at the wildlife-livestock interface (7, 11).

African swine fever (ASF) is a highly transmissible viral disease of swine that impacts global trade of swine and pork products. In Eurasia, ASF occurs in wildlife (wild boar; WB) and domestic pigs (DP) (8). In domestic pig populations, circulation is maintained through direct transmission between pigs within farms (12) as well as indirect transmission through fomites (e.g., contaminated feed, material, equipment) (13) or soft tick vectors in areas where the vectors exist (14). Proximity to infected farms and local density of DP are risk factors of farm ASF incidence in the Italian island of Sardinia (15, 16), Nigeria (17), Romania (18), Russia (19, 20), and globally (21). Between-farm transmission, involving transport of infected animals (direct transmission), equipment, feed and other fomites (indirect transmission), is closely related to trade and contact networks (22). For example, density of regional road networks is the most important risk factor for ASF occurrence in DP in Russia (19). Introduction of stringent regulations regarding domestic pig movements in the infected areas of the European Union (European Commission Implementing Regulation 2023/594) has reduced the risk associated with transport of live animals in relation to transmission through fomites.

In WB, ASF circulation is thought to occur through host-to-host contacts (direct transmission) and contaminated environments and infectious carcasses (indirect transmission). Patterns in ASF surveillance data in WB and modeling of ASF in WB suggests that the disease can persist endemically in the WB in some conditions (23–27). WB population density and habitat quality appear to drive patterns of ASF occurrence (26–30). High WB abundance enhances direct transmission (26), while carcass-based transmission is thought to be a key mechanism allowing low-level and long-term ASF persistence in WB populations, particularly at low densities (26, 29, 31). Studies in several different countries estimated an effective or basic reproduction number of ~1.5 between groups of WB from ASF surveillance data (11, 32–34) supporting the notion of endemic transmission levels in WB. However, these studies did not consider the potential role of DP in the estimates of effective reproductive numbers.

While DP and WB populations could each maintain ASF independently, bidirectional cross-cycle transmission is thought to occur (35). A primary mechanism of emergence of ASF in WB in new areas likely occurs through inappropriate disposal of infectious domestic pig carcasses or pork products in WB habitat followed by transmission among WB (12, 36). Once ASF occurs in WB populations, it is thought that the most likely routes of transmission from WB to DP is through contaminated feed or environments, and through direct contact depending on husbandry practices (12). Several studies have pointed to WB as an important risk factor for outbreaks in DP (13, 37), both in low-biosecurity backyard farms (18, 38) and high-biosecurity commercial farms (39). But, two important gaps remain: (1) determining if repeated transmission from DP to WB is important for explaining the patterns of detection in WB populations and, if so, how much transmission is important for persistence in WB (26, 40), and (2) whether transmission from WB to DP occurs and, if so, at what frequency. For example, Lange et al. (40) found little evidence of spatio-temporal clustering of WB detections suggesting lack of autonomous persistence in WB populations while Podgórski et al. (27) found substantial evidence for spatio-temporal clustering of detections in WB suggesting endemic transmission within WB populations. Pepin et al. (26) found autonomous persistence was likely in WB but depended on WB density and the frequency of carcass-based transmission, but this study did not include the potential for transmission from DP throughout the study area.

Few studies have examined surveillance data from WB alongside DP (e.g., 20, 32, 39) making it difficult to understand the occurrence and drivers of transmission dynamics among these host populations. While transmission from WB has been often implicated in ASF outbreaks in DP (18, 39), transmission from DP to WB has been rarely studied (20). Here, we integrate ASF surveillance data from WB and DP populations to better understand potential transmission between these host contexts. Our main objectives were to characterize risk factors of ASF occurrence in WB and DP populations and determine the relative frequency of transmission in each direction: WB-to-DP vs. DP-to-WB. We addressed these objectives by considering risk for WB and DP separately using covariates from the other population. We expected to observe greater transmission risk from WB to DP than the reverse based on the numerous reports of transmission risk from WB to DP, widespread occurrence of detections in WB, and free-roaming lifestyle of WB. Our analysis highlights important considerations for surveillance design at the wildlife-livestock interface.

## 2. Materials and methods

### 2.1. Surveillance data

ASF surveillance in Poland is regulated by international and national legislation [e.g., European Commission Implementing Regulation (EU) 2021/605 of April 7, 2021]. Intensity of surveillance and control measures follows a zoning system of restricted areas: zone III (ASF in DP and WB), zone II (ASF in WB), zone I (ASF high risk area, bordering zone II or III). Obligatory testing of all hunted (active surveillance), found dead, and road-killed (passive surveillance) WB is implemented in zones I (PCR test), II and III (PCR and ELISA/IPT tests), where our study area is contained (41). DP are tested in zones I, II and III (PCR) when moved from the holding to abattoir (all animals) and randomly within the holding (number of animals sampled scaled by holding size according to “Procedure for collecting and sending samples for laboratory diagnostics for African swine fever,” Chief Veterinary Officer, Poland, April 2020). Samples collected by veterinary services and hunters were analyzed by the National Reference Laboratory for ASF diagnostics at the National Veterinary Research Institute in Puławy, Poland. All positive results were confirmed by the European Reference Laboratory for ASF in Valdeolmos, Spain. We used surveillance data collected in 2014–2019 which totaled 1,244,117 test results of DP (including 2,184 ASF-positive results from 261 focal outbreaks, i.e., pig holdings) and 196,800 test results of WB (including 9,213 ASF-positive results, i.e., individual cases). Geographic coordinates were available for all domestic pig samples and positive WB samples. Negative WB samples were available aggregated at the level of commune, the smallest administrative unit in Poland. To make this subset of data compatible with the rest, we created a number of locations equal to negative WB tests in a given commune and assigned them randomly-generated geographic coordinates.

### 2.2. Data processing

**Figure 1:**
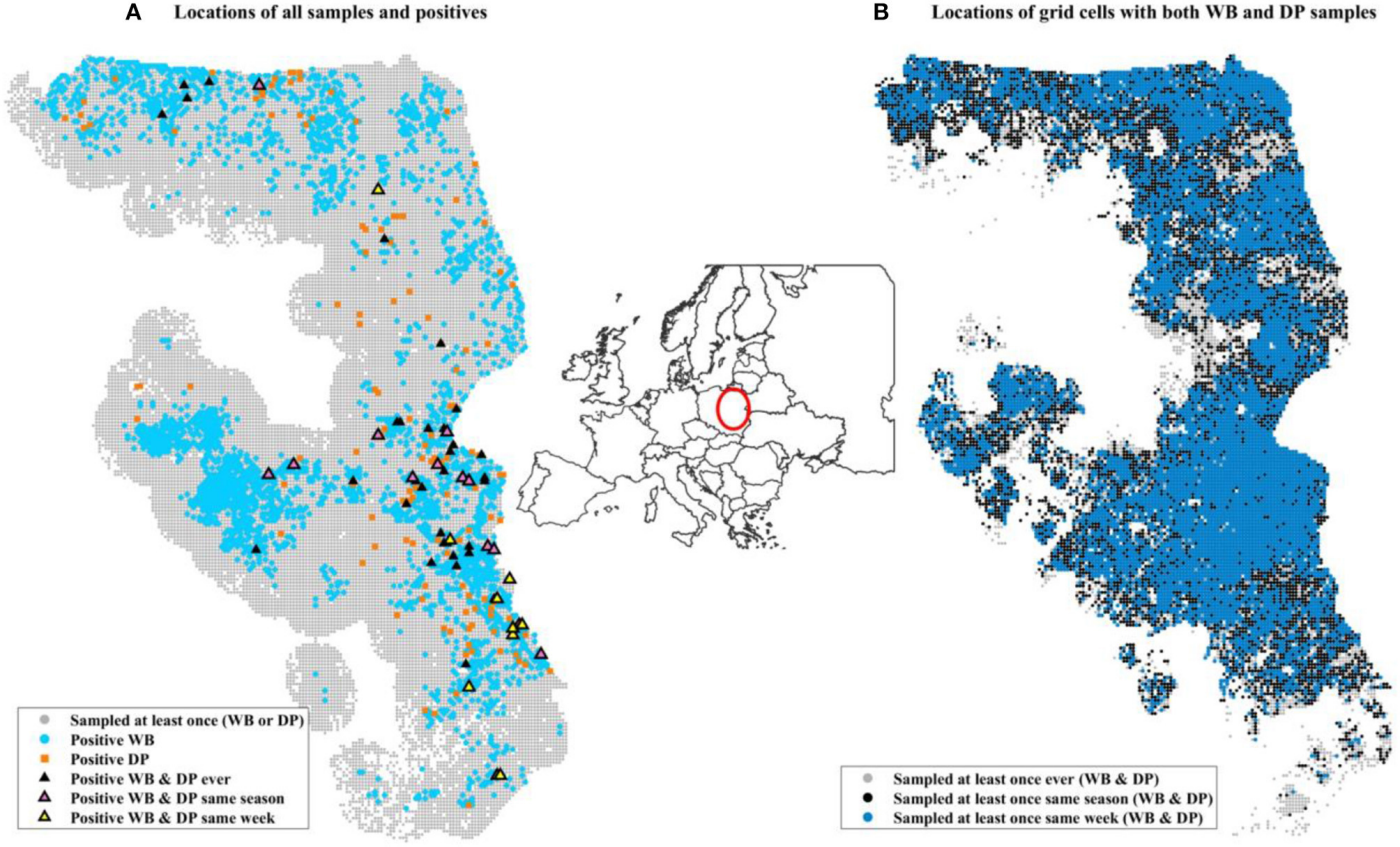
Maps of data included in the analysis. The inset map of Europe illustrates the location in Poland of the surveillance data using a red circle. **(A)** Includes all grid cells within 20 km (Euclidian distance) of a grid cell that was positive at some point during 2014–2019 (the entire time frame of our surveillance data). Small gray circles indicate a grid cell that was sampled at least once but never found to be positive. Light blue circles are locations of at least one positive WB sample during the 6 years. Orange squares are locations of at least one positive domestic pig. Black triangles are grid cells where at least one positive WB and one positive domestic pig was found at some point during the 6 years, not necessarily during the same time. **(B)** Shows the sampling design for grid cells that had sampling of both WB and DP at different time scales. The three colors distinguish the time frames within which at least one sample from each of WB and DP was collected: ever (gray, 106,247 unique grid cells), same season (black, 86,336), same week (blue, 7590 unique grid cells). The distribution of WB and DP samples collected in the same week (blue) is similar to that over broader temporal scales (gray and black).

Surveillance data (number of positives by PCR and number of samples collected) were aggregated at a weekly scale on a 2 km by 2 km grid cell resolution across all of Poland. Only grid cells that were ever positive themselves during the time frame of surveillance (2014–2019) or within 20 km of a positive were included in the analysis to control for biased weighting of landscape covariates that were never in the vicinity of disease. We also excluded an isolated western cluster in Lubuskie Voivodeship because it was small and contained and not thought to be involved in driving the dynamics in the eastern side of Poland (42). This resulted in 516,105 grid-cell-by-week combinations of data. DP samples were collected in 403,813 grid-cell-by-week data points, while WB samples were collected in 127,835 grid grid-cell-by-week data points (Table 1). There were 21,136 unique grid cells with at least 1 surveillance sample of any kind, and 15,153 of these unique grid cells had at least 1 surveillance sample collected from each of DP and WB during the 6 years of data (Table 1, Figure 1).

**Table 1:** Number of data points within 2 × 2 km grid cells for different temporal aggregations.

**Aggregation**	**Any sample**	**≥1 WB sample**	**≥1 DP sample**	**≥1 WB & 1 DP sample**	**≥1 WB positive**	**≥1 DP positive**	**≥1 WB & 1 DP positive**
2 × 2 km grid cell by serial week	516,105	127,835	403,813	16,121	5,457	279	12
2 × 2 km grid cell by year and season	152,405	91,873	94,975	34,642	3,979	191	23
2 × 2 km grid cell across all time	21,136	20,552	15,727	15,153	2,881	182	55

The independent variables used in the analyses, rationale for including them, and data sources are presented in Table 2. The response variable (binary) was the presence of positive samples within grid cell *k* in week *t* – a 0 if none of the samples in grid cell *k* in week *t* were positive and a 1 if at least one sample in grid cell *k* at week *t* was positive. We analyzed the data at different temporal scales of aggregation (week, season, or over all time) because timescales of disease persistence in carcasses remain poorly understood and we wanted to examine how the effects of covariates depended on the temporal aggregation of the response data. For the weekly aggregation, we created 4 response variables that we analyzed separately: all samples from WB (WB full model), all samples from DP (DP full model), samples from WB that occurred within 5 km of a PCR+ sample in DP in the last 4 weeks (WB submodel), and samples from DP that occurred within 5 km of a PCR+ sample in WB in the last 4 weeks (DP submodel). The last two responses allowed us to test for potential factors driving transmission between host populations (WB submodel, DP submodel) without noise from data points that were too far away to be linked to transmission. We chose 5 km distance because between-sounder contact and transmission is most likely within this distance (45, 46). For the season aggregation, we summed surveillance data for 4 separate seasons as specified in Table 2. Finally, we analyzed the data by summing over all time, thus only effects of spatial covariates were tested.

**Table 2:** Covariates used in the analyses.

**Variable**	**Description and mechanism**	**Processing**	**References for source data**
Area developed	Description: surface of the developed area in each grid cell Mechanism: drives contact rates between WB and DP	Total surface area of all built-up and developed areas within the cell (km^2^)	Topographic Objects Database (BDOT10k), Head Office of Geodesy and Cartography (https://bit.ly/3Ji1Mdh)
Human population density	Description: density of human population in each grid cell Mechanism: contamination between WB and DP through humans as a vector	Each grid cell was assigned with a density of human population (ind/ km^2^) from the commune (mean size of 126.2 km^2^) the cell was in	Statistics Poland (https://stat.gov.pl/en/)
WB habitat suitability	Description: quality of available habitats (QAH) based on global land cover vegetation (GLOBCOVER) Mechanism: higher quality habitats will sustain higher WB numbers and drive transmission	Average value of QAH was assigned to each grid cell. Input database categorized QAH into 7 levels at 300 × 300 m resolution.	(28)
Extensive farming of DP (more land to increase yield – e.g., holdings at lower density and biosecurity over more land)	Description: density of pigs bred in an extensive system. Mechanism: extensive pig farming facilitates transmission between WB and DP	Pig density (ind./km^2^) assigned to each grid cell from the FAO database available at the 5 minutes of arc (49.3 km^2^)	Food and Agriculture Organization (FAO) Gridded Livestock of the World; Global pigs distribution in 2015 (43, 44)
Hunter harvest	Description: number of WB harvested in each year and each grid cell. Mechanism: high WB numbers and hunting activity drive transmission	Each grid cell was assigned a hunting bag from the hunting ground the cell was in (mean size of a hunting ground was 6128 km^2^).	Forest Data Bank (https://bit.ly/3WDVlnJ)
Season	Description: Season that the sample was collected. Mechanism: transmission is higher at particular times of the year.	A level 1–4 was assigned to each data point. 1) December–February (weeks 49–53, 1–9) 2) March–May (weeks 10–22) 3) June–August (weeks 23–35) 4) September–November (weeks 36–48)	See Processing step
Sample size	Description: Number of surveillance samples submitted. Mechanism: Affects detection probability.	The count of surveillance samples submitted by grid and week for the response variable	Derived from surveillance data
Prevalence in neighborhood DP	Description: Recent prevalence in DP in neighboring grid cells. Mechanism: Proximity to infectious individuals drives infection.	The number of PCR+ samples from DP within the last 4 weeks within 5 km of the grid cell divided by the total number of samples in the same time/space scale.	Derived from surveillance data
Prevalence in neighborhood WB	Description: Recent prevalence in WB in neighboring grid cells. Mechanism: Proximity to infectious individuals drives infection.	The number of PCR+ samples from WB within the last 4 weeks within 5 km of the grid cell divided by the total number of samples in the same time/space scale.	Derived from surveillance data
Closest positive in DP	Description: Closest distance of recent positive detections in DP. Mechanism: Proximity to infectious individuals drives infection.	Minimum distance between a focal grid cell and the location of a PCR+ domestic pig sample within the last 4 weeks.	Derived from surveillance data
Closest positive in WB	Description: Closest distance of recent positive detections in WB. Mechanism: Proximity to infectious individuals drives infection.	Minimum distance between a focal grid cell and the location of a PCR+ WB sample within the last 4 weeks.	Derived from surveillance data

All variables were continuous except for season which was categorical variable with 4 levels.

### 2.3. Analyses

All analyses were conducted in Matlab R2021b (The Mathworks Inc, Natick, Massachusetts) using the Statistics and Machine Learning Toolbox. We modeled the data using boosted regression trees with a least-squares boosting loss function. We chose this approach because we expected complex interrelationships among independent variables in their effects on the response data and non-linear relationships. We optimized hyperparameters for each regression ensemble model using Bayesian optimization and 10-fold cross-validation aimed at minimizing the mean squared error implemented in fitrensemble using the Optimize Hyperparameters option. We co-optimized the following hyperparameters using the following specified prior ranges: number of learning cycles [50, 2000] (except DP week full model was [50, 1000] for computational feasibility), learning rate [0.0001, 0.1] (except DP week full model was [0.001, 0.1] for computational feasibility), minimum leaf size [50, 100], maximum number of splits [1, 60] (Table 3). The small range on maximum number of splits and high minimum value on minimum leaf size was chosen to reduce overfitting. The optimization was run for 5000 iterations on each data set except for the DP full model that was run for only 1000 iterations (because the dataset was very large and preliminary runs showed early convergence).

**Table 3:** Optimal hyperparameters for each model.

**Model**	**N**	**PCR+**	**Total iterations**	**Iteration where lowest MSE was reached**	**Optimal hyperparameter estimates**			
					**Learning rate**	**Learning cycles**	**Minimum leaf size**	**Maximum number of splits**
WB week submodel	792	194	5000	151	0.0084	1968	50	2
DP week submodel	45,128	141	3000	1,459	0.1000	1934	94	15
WB week full model	127,835	5,457	3000	133	0.0998	106	69	59
DP week full model	403,813	279	1000	562	0.0983	980	62	27
WB season	91,873	3,979	3000	318	0.0990	50	100	43
DP season	94,975	191	3000	1,565	0.0515	361	50	31
WB all time	20,552	2881	3000	535	0.0058	651	93	55
DP all time	15,727	182	3000	931	0.0962	931	97	2

N is the number of data points in the model; PCR+ are the number of samples that tested positive for ASFv with the PCR assay.

We then fit the final models with the optimal hyperparameters to estimate variable importance and make inferences about the effects of independent variables on the responses for each model.

## 3. Results

### 3.1. Spatial and temporal trends in cases

Of the 15,153 unique grid cells that had at least one surveillance sample from each of WB and DP during the 6 years of surveillance, 2,881 cells had at least one positive WB sample, while only 182 cells had at least one positive DP sample (Table 1). Only 55 grid cells found at least one positive WB and DP sample in the same grid cell during the 6 years of surveillance. Of the 16,121 surveillance data points that involved collection of at least 1 WB and 1 DP sample in the same grid cell on a given week, 5,457 grid cell-by-week data points had at least one WB positive sample, while only 279 had at least one positive sample for DP. There were only 12 grid-cell-by-week data points and 23 grid-cell-by-season data points that had at least 1 positive sample for each of WB and DP samples (Table 1, Figure 1). Thus, given the wide spatial extent of WB positive samples (2,881 unique grid cells), and numerous data points where at least one WB and one DP sample were collected in the same grid cell in the same week (16,121), most WB positive samples did not temporally overlap with the unique grid cells where positive DP samples were found. Only 6.6% (12/182) occurred in the same grid cell in the same week, 12.6% (23/182) occurred in the same grid cell in the same season, and 30% (55/182) occurred in the same grid cell ever (across 6 years).

Of the 279 grid-cell-by-week data points that had at least one DP positive sample, there were only 61 (21.8%) with at least one surveillance sample from WB within 5 km of the grid cell within the last 4 weeks, and only 15 of those instances (25% of WB samples, 5.4% of the DP-positive grid-cell-by-weeks) had at least one positive WB sample. There were 217 DP-positive grid cells that had at least 1 DP surveillance sample in the vicinity (77.8%), but only 10 of those had a DP-positive sample (10/217 = 4.6% of DP-positive samples with DP surveillance samples or 3.6% of the total DP-positive grid-cell-by-weeks). Thus, the large majority of DP positive surveillance data did not include surveillance in WB nearby, despite including DP surveillance nearby, but when surveillance did occur nearby it was more likely that the WB samples were positive relative to the DP samples.

In contrast, of the 5,457 grid-cell-by-week data points that had at least one WB positive sample, 2,287 (41.9%) had at least one surveillance sample from DP within 5 km of the grid cell within the last 4 weeks, but only 12 (0.52% of DP samples, 0.22% of the WB-positive grid-cell-by-weeks) had at least one positive surveillance sample from DP. Thus, the surveillance design afforded twice as much opportunity to find DP-positive samples in the recent vicinity of WB-positive samples compared to the opposite (compare 41.9 to 21.8%), yet the reverse occurred – the rate of positive WB samples in the recent vicinity of a positive DP sample was 48 times as likely than the rate of positive DP samples in the recent vicinity of a positive WB sample (divide 25% for WB-positive samples collected in the recent vicinity of a DP positive by 0.52% of the DP-positive samples collected in the recent vicinity of a WB-positive sample).

**Figure 2:**
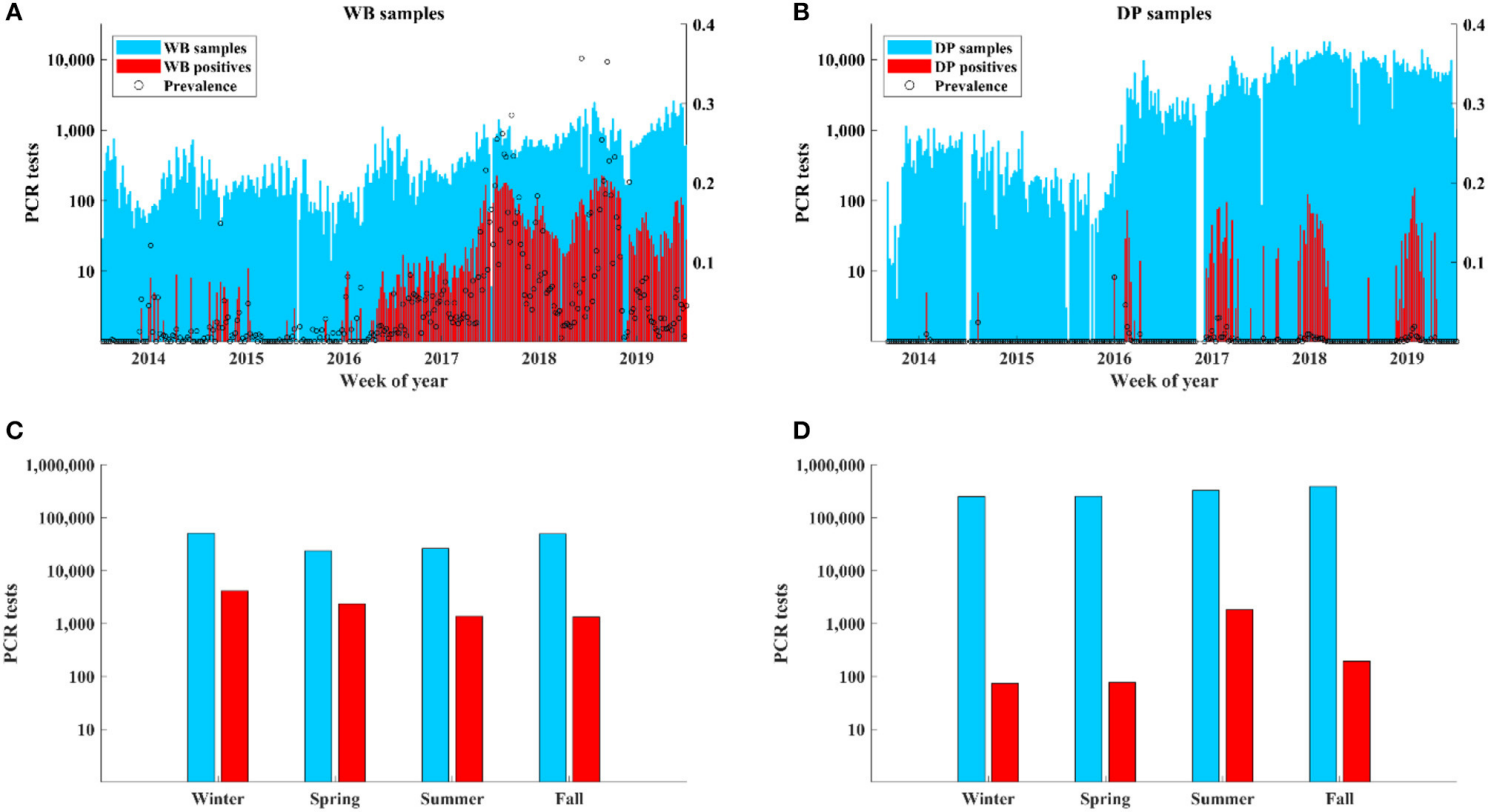
ASF surveillance data over time.

Cases in WB were detected throughout the year at similarly high levels with the highest detection rates occurring in winter (45%; 4150/9198) and spring (25%; 2351/9198) relative to summer (14.9%; 1366/9198) and fall (14.5%; 1331/9198) (Figures 2A, C). In contrast, cases in DP showed a distinct seasonality with most cases (84.2%; 1832/2174) occurring in summer despite a similar number of samples being collected during each season with the most being collected in the fall (Figures 2B, D).

### 3.2. Correlation with potential risk factors

**Figure 3:**
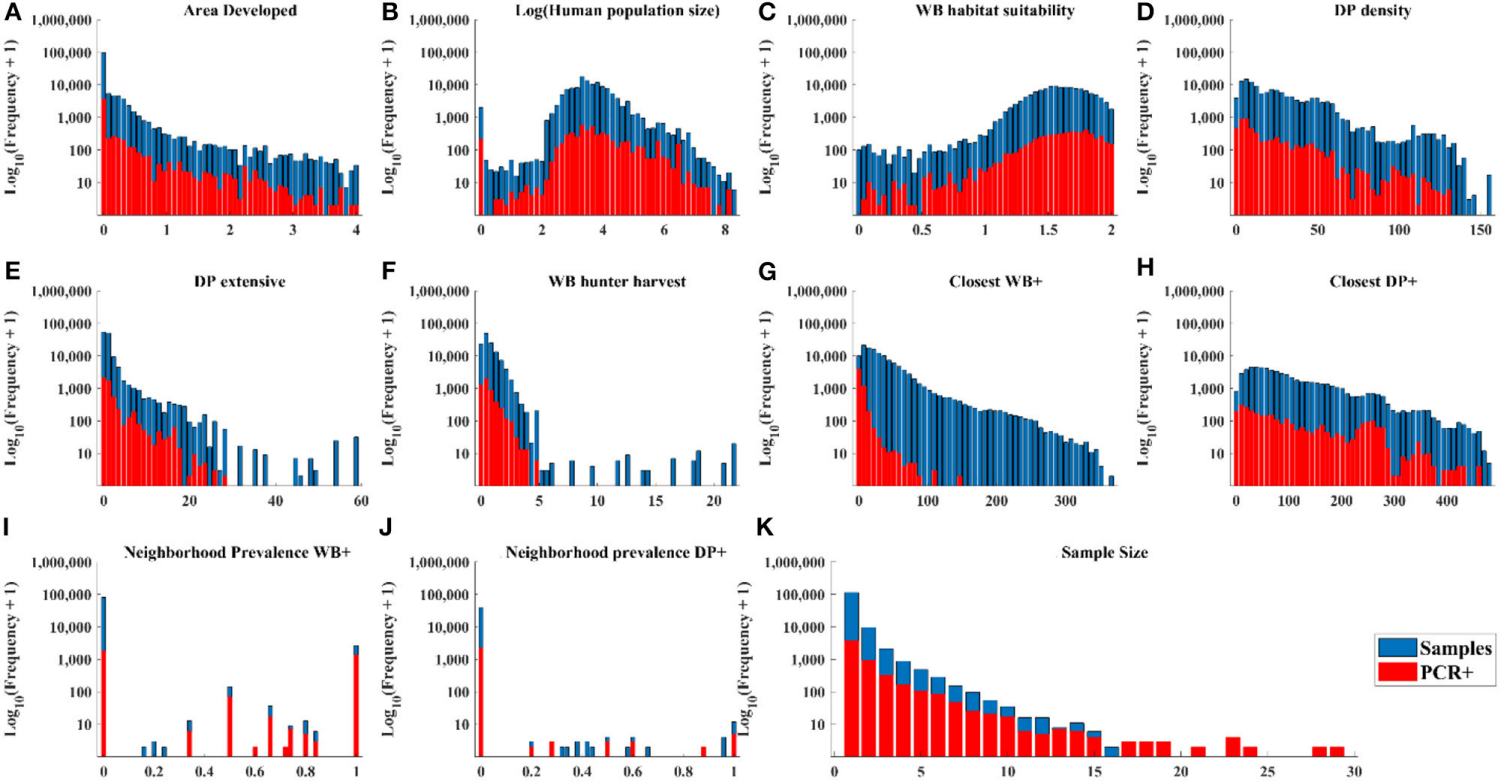
Distribution of WB samples and positive tests by PCR within the range of each independent variable.

**Figure 4:**
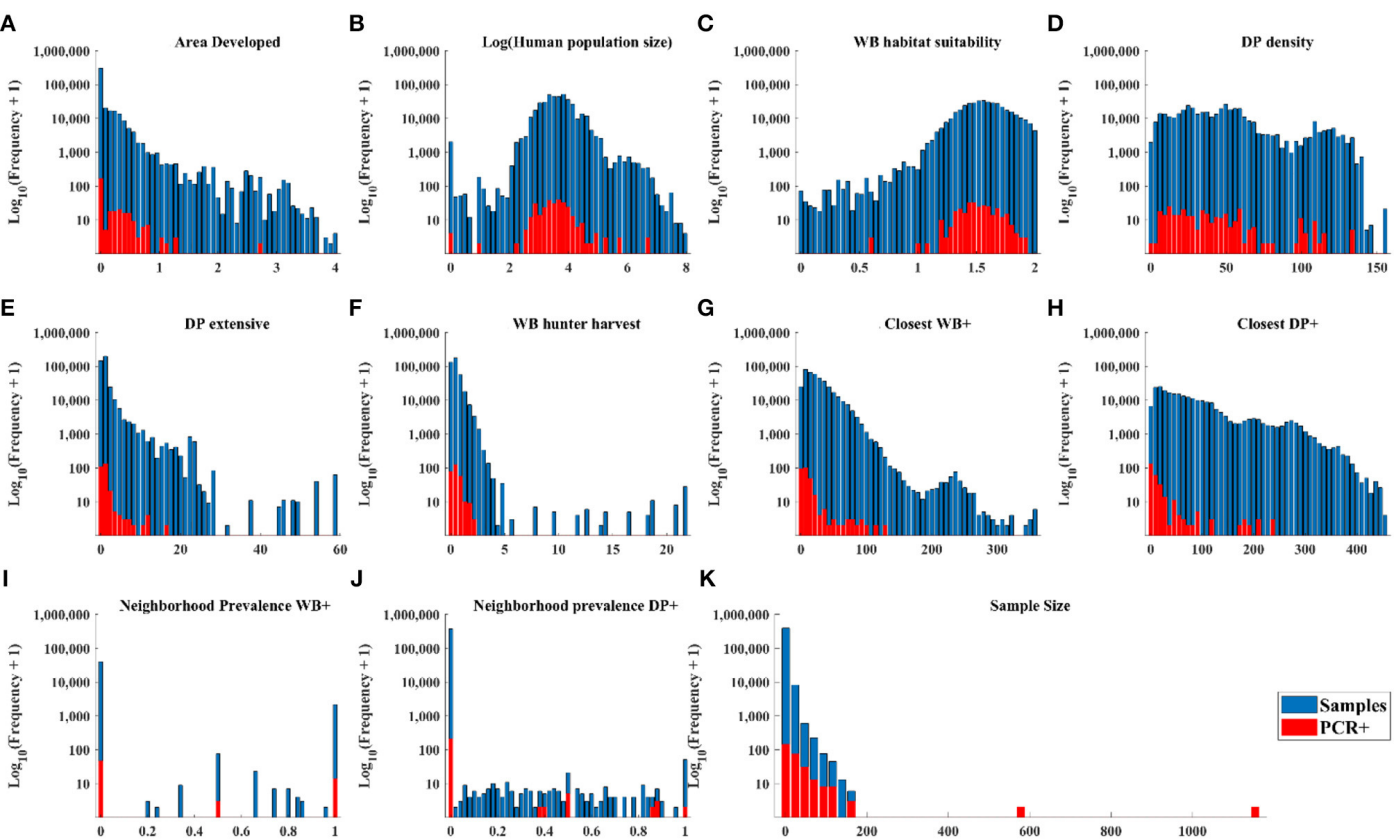
Distribution of DP samples and positive tests by PCR within the range of each independent variable.

The distribution of cases in WB tracked the distribution of independent variable values closely (Figures 3A–D, H, J) except there were: (1) a small number of very high values for DP extensive and hunter harvest where WB cases were not found (Figures 3E, F), (2) visible trends of more cases at closer distances of recent cases in WB and higher neighborhood prevalence in WB (Figures 3G, I), and (3) visible trends of higher prevalence at larger sample size (Figure 3K). The distribution of cases in DP showed similar trends relative to independent variables except that cases clustered toward the closer distances of cases in both WB and DP (Figure 4).

### 3.3. Risk factors involving DP were not important for predicting ASF detection in WB

**Figure 5:**
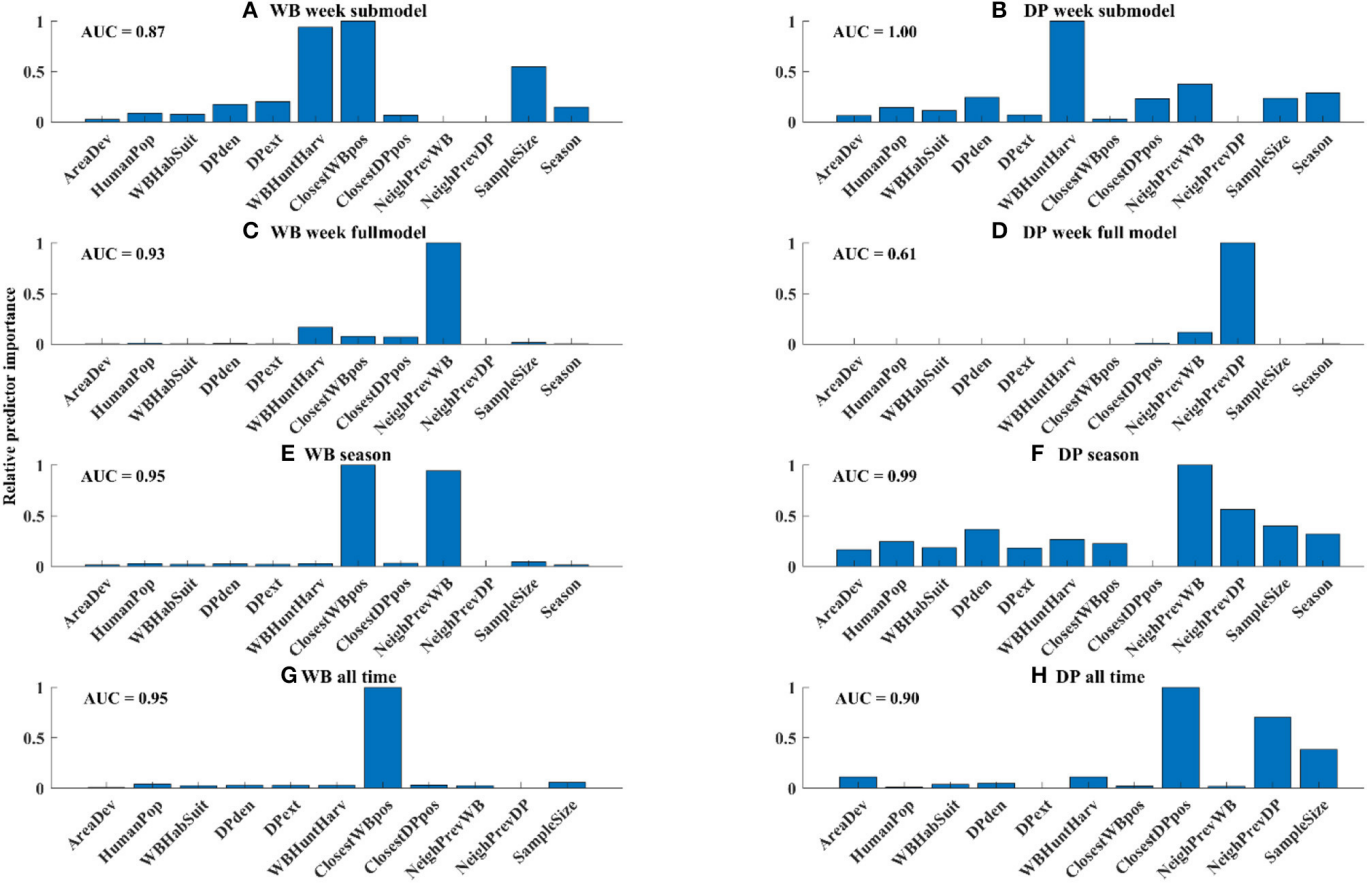
Relative variable importance for each model. Absolute goodness-of-fit for each model was measured by AUC and is shown in the upper left of each panel.

In the WB week submodel, which limited the data only to WB samples that occurred within 5 km of a PCR+ sample in DP in the last 4 weeks, the number of WB harvested and closest distance to the nearest WB-positive detection were the most important risk factors, followed by sample size (Figure 5A). However, when all the WB samples were considered in the model, the only important risk factor of WB positivity was the neighborhood prevalence in WB (Figure 5C). When aggregating the data at seasonal scale both the neighborhood prevalence in WB and distance to the nearest WB sample were important risk factors (Figure 5E), whereas when all data was pooled, only distance to the nearest WB sample was an important risk factor (Figure 5G).

### 3.4. Risk factors involving WB were important for predicting ASF detection in DP

In the DP week submodel, which limited the data only to DP samples that occurred within 5 km of a PCR+ sample in WB in the last 4 weeks, the number of WB harvested (a proxy for WB density) was the most important risk factor, followed by distance to the closest DP-positive sample, recent neighborhood prevalence in WB, sample size, and season (Figure 5B). However, in the full model for DP, the most important variable was neighborhood prevalence of DP, followed by neighborhood prevalence of WB (Figure 5D), although the best full model did not fit the data very well (AUC = 0.61). When aggregating the data to the season scale neighborhood prevalence in WB was the strongest risk factor, followed by neighborhood prevalence in DP and sample size (Figure 5F), whereas when the data were aggregated across all time, only proximity to DP-positive samples was an important risk factor (Figure 5H).

## 4. Discussion

Our analysis revealed surprisingly few surveillance data containing samples for both WB and DP (12.6% for WB and 4.0% DP data) on spatial and temporal scales that are most relevant to transmission (e.g., within 4 km^2^ and the same week). This emphasizes that active surveillance of WB around DP premises might be needed in addition to passive surveillance to understand transmission routes and frequency between WB and DP host populations from ASF surveillance data. We addressed the surveillance design issues by examining effects of risk factors across a variety of spatial and temporal scales. At all scales, positive samples in WB were predicted by WB-related risk factors (e.g., recent proximity to WB-positive samples, hunter-harvest samples - a proxy for WB density, WB sample size), but not to DP-related risk factors. In contrast, WB risk factors were important for predicting detections in DP on a few spatial and temporal scales. These trends occurred even though the sampling design afforded more opportunity to detect DP as a risk factor of ASF detection in WB relative to the ability to detect WB as a risk factor of ASF detection in DP.

In several studies, the presence of ASF-infected WB in close vicinity to DP holdings has been cited as a main risk factor for ASF outbreaks in DP (47). Our results are consistent with these findings. However, we also addressed the gap (48, 49) of whether DP pose a risk of transmission to WB. Our results in eastern Poland suggested that the WB were almost 50 times more likely to pose a transmission risk to DP than the other way around. Experimental infection data show that WB and DP are equally susceptible to ASF virus when inoculated through similar routes (50) thus these differences are likely due to ecological or behavioral factors at the wildlife-livestock interface (51). For example, WB are social and disruptions of/in social structure affects movement behavior (52). It is possible that in areas with wide circulation of virulent strains of ASF or high hunting pressure WB change their movement behavior and seek interaction with other swine, even DP (51), or escape disturbance (53, 54). This could lead to higher rate of transmission among WB and from WB to DP. Also, studies that investigated interaction frequency between WB and DP have documented a wide range of interaction rates depending on the husbandry practices, biosecurity levels, and ecological context (49, 51, 55). Some of these studies documented high rates of direct contact while others mainly indirect contacts. It is also likely that indirect contact routes pose different transmission risks between WB to DP relative to the reverse. Accounting for variation within these contextual factors in analyses of ASF surveillance data would be valuable for quantifying the frequency of WB-DP transmission by different routes.

Similar to previous work we found that seasonal peaks of ASF detections in WB and DP were not synchronized (winter-spring for WB vs. summer for DP) (15, 56), but quantitative information on how much transmission varies seasonally is currently missing. Our results in eastern Poland suggest that 85% of transmission among DP occurred in summer, while only 15% of transmission in WB occurred in each of summer and fall. The low rates of detection in DP in fall and winter but high rates of detection in WB in winter further supports our finding of a low rate of transmission from DP to WB. In contrast, detections in DP were highest in summer, which follows the highest season of detections in WB and supports the higher frequency of transmission from WB to DP. It has been hypothesized that summer peaks in DP are driven by indirect transmission from the surrounding environment (through movements of contaminated feed, bedding, equipment during intensive field work) (39) while winter-spring peaks in WB are driven by seasonal factors, such as longer carcass persistence and birth pulses that introduce susceptible individuals.

The sampling design made it difficult to estimate the relative frequency of transmission from WB to DP and the reverse. One gap is that locations of negative WB samples are imprecise - georeferenced to the commune instead of GPS coordinates of collection. GPS coordinates for negative WB samples would allow for more precise estimates of the distance between DP and WB samples. It is important to understand the spatial sampling design for all samples for quantifying risk. Secondly, for DP premises with ASF outbreaks, it was uncommon for WB samples to be collected within 5 km, making it challenging to assess the potential role of WB in seeding DP outbreaks. Epidemiological investigations that coordinate veterinary and wildlife agencies in surveillance sampling around DP premises will help to better understand potential transmission routes between WB and DP. Relatedly, a mechanism for capturing husbandry practices or biosecurity actions around premises would provide additional information for inferring transmission routes. These gaps in surveillance design are not due to a lack of WB presence near DP. For example, there were > 16,121 grid-cell-by-week data points and 34,642 grid-cell-by-season data points that had at least one DP and one WB sample. Thus, it appears there is opportunity to conduct targeted surveillance in WB and DP populations around positive cases. Another valuable approach could be to conduct targeted risk-based surveillance where higher numbers of DP and WB samples would be collected on and around premises where outbreaks have a higher likelihood of occurring based on historical data or other risk assessments conducted at a fine spatial resolution (e.g., premises-level and within 5 km of premises). These surveillance approaches could be paired with other important metadata (i.e., husbandry and biosecurity practices).

A challenging gap to address is the poor understanding of long-range movements in both DP and WB populations. Our analysis does not address long-range connectivity in either WB, DP, or their interface. Most WB movement and contact is thought to be close (45) but there may be some longer distance WB movements (57) or other mechanisms (fomites, contaminated meat products) that spread ASF over longer distances in WB (42). However, DP may be moved over longer distances more regularly. Data describing these movements are important for quantifying the role of WB in outbreaks in DP and potential for the reverse.

## Data availability statement

The raw data supporting the conclusions of this article will be made available by the authors, without undue reservation.

## Author contributions

KP: Conceptualization, Formal analysis, Investigation, Visualization, Writing—original draft, Writing—review & editing. TB: Data curation, Investigation, Writing—review & editing. MF: Data curation, Writing—review & editing. KP: Data curation, Writing—review & editing. TP: Conceptualization, Data curation, Investigation, Visualization, Writing—original draft, Writing—review & editing.
